# Impact of noise on hearing in the military

**DOI:** 10.1186/s40779-015-0034-5

**Published:** 2015-02-25

**Authors:** Jenica Su-ern Yong, De-Yun Wang

**Affiliations:** Department of Otolaryngology-Head and Neck Surgery, National University Health System, National University of Singapore, Singapore, Singapore

**Keywords:** Hearing loss, Noise-induced, Military personnel, Ear protective devices

## Abstract

Hearing plays a vital role in the performance of a soldier and is important for speech processing. Noise-induced hearing loss is a significant impairment in the military and can affect combat performance. Military personnel are constantly exposed to high levels of noise and it is not surprising that noise induced hearing loss and tinnitus remain the second most prevalent service-connected disabilities. Much of the noise experienced by military personnel exceeds that of maximum protection achievable with double hearing protection. Unfortunately, unlike civilian personnel, military personnel have little option but to remain in noisy environments in order to complete specific tasks and missions. Use of hearing protection devices and follow-up audiological tests have become the mainstay of prevention of noise-induced hearing loss. This review focuses on sources of noise within the military, pathophysiology and management of patients with noise induced hearing loss.

## Introduction

Noise-induced hearing loss is a major preventable disease. It can be caused by an acute exposure to an intense impulse of sound or by a continuous steady-state long-term exposure with sound pressure levels higher than 75–85 dB (Table 1).

**Table 1:** **Glossary of terms used**

**Terms**	**Descrption**
**Sound pressure level (SPL)**	Sound intensity is expresses the pressure caused by a sound wave and is indicated by sound pressure level. The unit of measurement is the decibel (dB SPL)
**dB Scale**	A logarithmic scale to measure sound pressure level
**dBA**	To measure noise, A-weighted SPL (dBA) can be used. In contrast to SPL which represents a physical dimension, A-weighted SPL represents a perceptual dimension. The dB SPL will be different from dBA for different for different frequencies as low frequency sounds and high frequency sounds tend to be less loud than mid-frequency sounds
**L**_**Aeq**_	This refers to the average level of sound pressure within a certain time period with the A-filter used for frequency weighting. The A-filter is a frequency-weighting of sound pressure levels that mimics the sensitivity of the auditory system of humans (eg, low-frequency sounds contribute little to the A-weighted dB level)

Noise remains a large public health problem with an estimated 1.3 billion people being affected by hearing loss [1]. It ranks 13th globally as the cause of years lived with disability (YLD). YLD is estimated by multiplying the number of incident cases in that period with the duration of disease and the weight factor which measures disease severity. In North America, it ranks 19th as the cause of YLD, in Central Asia, it ranks 15th and in Southeast Asia it ranks 9th.

The prevalence of hearing loss and tinnitus in military population are greater than in the general public. Almost every soldier, sailor, airman or marine will be exposed to hazardous noise levels at some point in their career [2-4]. The two most prevalent service connected disabilities for veterans in the United States at the end of fiscal year 2012 remain tinnitus and hearing loss, with tinnitus affecting 115,638 veterans (9.7%) and hearing loss affecting 69,326 veterans (5.8%) [5]. In Finland, despite the increasing use of hearing protection devices, a large proportion of professional soldiers experience disabling tinnitus and hearing loss [6].

Hearing acuity is a key component of a soldier’s effectiveness in the battlefield. The presence of tinnitus and hearing loss can significantly impair a soldier’s ability to hear important acoustic cues or communication signals from the unit or the enemy [2]. Hearing problems can also be a reason for disruption of their military service. In a study by Muhr et al., 33 soldiers (3.9%) had interrupted training as a result of their hearing problems [7].

## Review

### Sources of noise-induced hearing loss

#### Land force

Sources of noise within the military vary with soldier’s designation. Within the Belgian military, Fighting in Built-Up Area (FIBUA) training, shooting with large calibre weapons and participation in military exercises were the strongest determinants of hearing loss [4].

Within the infantry, weapons emit high levels of noise. Table 2 depicts the amount of permissible noise allowed and Table 3 depicts the typical noise level emitted by different weapons. Many weapons emit sounds that exceed the maximum achievable protection that double hearing protection can offer. Double hearing protection means both earmuffs and ear plugs are used. The US Department of Defense published a medical surveillance monthly report on noise-induced hearing loss and it was found that noise-induced hearing injuries were more prevalent among combat-specific occupations (41.2 per 1000 person-years of active component military service) [8].

**Table 2:** Amount of permissible noise exposure allowed in theworkplace*

**Duration per day (hour)**	**Sound level (dBA)**
8	90
6	92
4	95
3	97
2	100
1 ½	102
1	105
½	110
¼ or less	115

*Adapted from OSHA 2014. Standards. US Dept Labor: Occupational Noise Exposure [Online]. vailable by Occupational Safety and Health Administration. https://www.osha.gov/SLTC/noisehearingconservation/index.html.

**Table 3:** Peak sound pressure level range of different weapons*

**Type of weapons**	**Peak sound pressure level range (dB)**
**Rifles**	
.45-70 Rifle	155.2-159.9
.30-06 Rifle	158.7-163.1
**Shotguns**	
.410 Bore	151.0- 157.3
20 Gauge	154.8
12 Gauge	156.1- 161.5
**Pistols**	
.22	151
9 mm Luger	159 163
.45 ACP	158
**Other Weapons**	
Hand grenade	158
Light anti-tank weapon	184
Inside armored vehicle, continuous noise	L_Aeq_103 – 107

*Adapted from Chen L, Brueck SE. Noise and lead exposure at an outdoor firing range – California. Health Hazard Evaluation report Sept 2011, and from Kramer WL. Gunfire noise and hearing. American Tinnitus Association. June 2002:14–15.

#### Navy

In the Navy, the highest indoor noise levels were found in engine rooms [9,10]. Landing ship tanks and patrol vessels typically generated about 98 to 103 dBA of noise, whereas the noise level in missile gun boats were at 120 dBA [9]. The loudest noise generated is on the carrier decks that can range from 130 to 160 dBA [2].

#### Air force

Military aircraft personnel are not spared, the average noise experienced in service helicopters was found to be 97 dBA for ‘Gazelle’ , 99.8 dBA for the ‘Scout’ , 99.9 dBA for the ‘Puma’ and 100 dBA for the ‘Lynx’ [11]. In fighter planes, the noise level ranged from 97 to 104 dBA, in jet trainers the noise level was at 100 to 106 dBA and in transporter aircrafts, the noise level was found to be between 88 to 101 dBA [12]. In such settings, due to chronic noise exposure, pilots were found to exhibit hearing impairment [13].

### Pathophysiology

Injury from noise can occur in 2 main ways. First, high level, short duration exposure exceeding more than 140 dB can cause the delicate inner ear tissues to beyond stretch beyond their elastic limits. This causes mechanical disruption of the sterocilia and direct damage to supporting and sensory cells [14]. In such cases, the maximum sound pressure level (SPL) is more important than the duration of the exposure [15]. This type of acoustic trauma can result in immediate and permanent hearing loss.

Second, long term exposure to low level noise damages the cochlea metabolically rather than mechanically. It involves biochemical pathways leading to cell death either through apoptosis or necrosis [16]. There are 2 factors that influence which cell death pathway is activated. The first factor is the sound intensity level. Noises of 105 dB favour necrosis whereas louder noises (120 dB) favour apoptosis [17]. Another factor is the time between noise exposure and morphological analysis. Outer hair cells immediately start dying during the initial acoustic insult and continue to do so for at least 30 days after the event [18,19]. Immediately after the insult, apoptosis is the main cause of cell death. After 4 days, the apoptotic activities start to diminish and by day 30 both apoptosis and cell necrosis contribute equally to cell death [19,20].

Exposure to intense sound can cause auditory thresholds to become elevated permanently or temporarily. Reversible hearing loss is referred to as temporary threshold shift (TTS). Depending on duration of exposure, recovery from TTS can occur over a period of minutes to hours or days. If TTS does not recover, permanent hearing loss results and this is referred to permanent threshold shift (PTS) [21]. These two phenomena, permanent and temporary threshold shifts are still not well understood.

PTSs are postulated to be either due to direct mechanical trauma or metabolic overstimulation of cellular elements within the organ of Corti which is associated with generation of reactive oxygen species [22].

Various mechanisms have been proposed for TTS and include synaptic fatigue, metabolic fatigue of either stria vascularis or hair cells and changes in cochlear blood flow. An important component of noise-induced hearing loss is postsynaptic damage in the afferent dendrites beneath the inner hair cells [23]. Even though hair cells recover normal function, there is rapid extensive and irreversible loss of synapses and delayed and progressive loss of cochlear neurons over many months [24,25]. This resultant cochlear neuropathy has been observed in mice exposed to just 84 dB SPL over a week [26]. It is possible that many people with difficulty in hearing also suffer from noise-induced cochlear neuropathy seen in animal studies.

Noise not only increases hearing threshold, but it can also cause tinnitus and hyperacusis. This can be present in individuals with normal hearing thresholds but with cochlear neuropathy. Indeed, studies have shown that patients with tinnitus have evidence of reduced Wave I at high sound levels [25,27]. The pathogenesis of tinnitus is postulated to be due to a compensatory increase in neural gain to the auditory brainstem as a result of reduced neural output from cochlea [27,28]. The gain can lead to tinnitus due to the amplification of spontaneous activity of auditory neurons.

### Clinical presentation

#### Symptoms and signs

Exposure to noise can induce several hearing symptoms such as temporary threshold shifts (TTS), tinnitus, hyperacusis, recruitment, distortion or abnormal pitch perception [29]. Tinnitus can occur in the presence or absence of an abnormal audiogram. The tinnitus pitch match is associated with the frequency spectrum of hearing loss [30,31].

Patients may exhibit difficulty in listening to high frequency noise such as whistles or buzzers. They may also have difficulty differentiating some speech consonants, especially if they are in areas where there is significant background noise.

However these symptoms are typically insidious and most patients with noise induced hearing loss may not notice their deficiency until it starts to affect communication.

### Audiometric characteristics

Noise-induced deafness usually occurs at high frequencies with hearing loss beginning around 4 kHz or 6 kHz. However, as the disease progresses, hearing loss will also be seen at the lower frequencies. The expected maximal changes in thresholds are predictable at one-half octave above maximal frequency of the exposure [32].

The audiometric pattern in noise induced hearing loss is usually symmetrical and bilateral. However some asymmetry is not unexpected. The asymmetry in hearing threshold may be partly explained by the position of head during work [33]. Hong et al. studied workers in the American construction industry and it was found that the left ear predominantly experienced more hearing loss than the right. Asymmetry was postulated to be due to the work habit that the operators look over their right shoulder when operating heavy equipment, exposing their left ear to the noise generated by the machines [34]. Hearing loss among rifle shooters also tend to be asymmetrical, as hearing in the ear closest to the barrel tends to be worse as it is closer to the explosion whereas the other ear is protected by the head [12,35]. In the civilian population, this was also seen in musicians who played high string instruments where the left ear was found to be exposed to 4.6 dB more than the right ear [36].

### Management of patients

#### Noise prevention

Within the military setting, noise exposure may be controlled through isolation (distance and physical barriers), vibration dampening, insulation and proper equipment maintenance [37]. The preferred method of preventing noise induced hearing loss and noise induced tinnitus is engineering controls. Other methods including the use of hearing protection devices such as foam ear plugs, molded insets and sound attenuating ear muffs are limited and can diminish perception of speech. Prevention is also reliant on the individual’s compliance to the sound protection devices.

Currently, the Navy considers 85dBA to be the threshold for single hearing protection and 104 dBA for double hearing protection for steady state noise settings [38]. Noise levels on the flight deck during flight and some aircraft maintenance operations are intense and can easily exceed the 104 dBA threshold for double hearing protection [2].

In the British Army Air Corps, pilots of the Lynx have to wear the Mk4 flying helmet and pilots of the Apache wear the Integrated Helmet and Display Sighting System (IHADSS). Circumaural earmuffs are integrated into the aircrew helmet system. Lang *et al.* found that hearing was better than predicted in nearly all frequencies for both ears for both Lynx and Apache pilots, demonstrating that the circumaural earmuffs implemented reduce the risk of noise induced hearing loss [39].

Even the best hearing protection equipment will be ineffective if it is not used properly or if soldiers are not compliant. A focus group study found that main concerns with hearing protection were interference with detection and localization of auditory warning and perception of orders [40]. Bjorn *et al.* conducted a study on the hearing protection equipment use by the crew on the flight deck and found that 79% of flight deck personnel received an estimated 0–6 dB rather than the expected 28–30 dB of noise attenuation from either misuse of earplugs or non-compliance to ear plugs [41].

### Pharmacotherapy

Currently there is no established treatment for patients and it is limited to prevention and follow-up. However recent clinical trials have proved promising.

### Magnesium

Magnesium efficacy was tested in a double-blind study. Test subjects were given either 122 mg of magnesium or a placebo for 10 days and thereafter subjected monoaurally to 90 dB SPL of white noise for 10 minutes. TTS of > 20 dB was found in 28% of the placebo group compared to 12% in the magnesium-supplemented group [42].

Attias *et al.* conducted a double-blind placebo controlled study on army recruits and concluded that recruits who had magnesium supplementation had less frequent noise-induced PTS compared to the placebo group [43]. These 300 army recruits underwent basic military training where they were subjected to shooting range noises of an average peak level of 164 dBA and <1 ms duration with the use of ear plugs which reduced noise level by about 25 dBA. PTS was defined as a threshold >25 dB hearing loss in at least 1 frequency and it was found that PTS was higher in placebo group (11.5%) as opposed to the participants in the magnesium group (1.2%).

### N-acetyl-cysteine (NAC)

NAC acts as a reactive oxygen species scavenger and is postulated to reduce noise-induced hearing loss by reducing the exposure of the cochlea to reactive oxygen species. Glutathione S-transferases (GST) are a family of detoxification enzymes which help cells resist oxidative injury. Glutathione detoxification can be affected in individuals with genetic polymorphisms involving deletion of base pairs in the genes like GSTT1 and GSTM1. Patients with these two high-risk genotypes are more prone to have oxidative injury from noise induced hearing loss [44,45]. In a trial conducted on steel manufacturing workers, employees were administered either 1200 mg of NAC or placebo. Trial was conducted in a 2 × 2 crossover design with subjects taking either NAC or placebo for 14 days and with a 14-day wash-out period between treatments. Noise exposure was 88.4 - 89.4 dB as assessed by personal noise monitoring. The difference between the TTS was not found to be significant. However, when the subjects were subdivided based on genetic polymorphisms or GSTT1 and GSTM1, the subgroup with null genotypes in both GSTT1 and GSTM1 experienced protection by NAC [46].

### Methionine (MET)

Another glutathione (GSH) precursor is MET, an essential amino acid that can be converted to cysteine, which is the rate-limiting substrate for GSH production. It has been shown in animal studies to be otoprotective when administered at 200 mg/kg [47]. A major limitation in human studies are high-doses administration, route of administration and bioavailability.

### Ebselen

Ebselen is a potent glutathione peroxidase mimic and neuroprotectant. It also has strong activity against peroxynitrite, a super reactive oxygen species [48,49]. It reduces cytochrome c release from mitochondria and nuclear damage during lipid peroxidation [50]. Since it acts as a catalyst, low does maybe sufficient to prevent or treat noise induced hearing loss [51]. Phase II trials are currently in progress to determine the efficacy of oral ebselen.

## Conclusion

Noise-induced hearing loss is a serious disease burden in the military. Due to the nature of the military profession, hearing is a vital asset during tactical and survival training and exposure to loud noises during training and missions are inevitable. Prevention is still the mainstay of treatment and soldiers need to be educated with regards to the use of hearing protection devices.
